# Primary Leiomyosarcoma of Bone: Analysis of Prognosis

**DOI:** 10.1155/2012/636849

**Published:** 2012-03-11

**Authors:** P. Brewer, V. Sumathi, R. J. Grimer, S. R. Carter, R. M. Tillman, A. Abudu, L. Jeys

**Affiliations:** Department of Orthopaedic Oncology, The Royal Orthopaedic Hospital, Birmingham B31 2AP, UK

## Abstract

Leiomyosarcoma of bone is just one of the variants of spindle cell sarcoma of bone characterised by the expression of desmin and other markers indicating a significant element of smooth muscle in the tumour, without osteoid production we have investigated the management and outcome of this rare type of primary malignant bone tumour. *Method*. Retrospective review of data stored on a prospective database. *Results*. In a database of 3364 patients with primary malignant bone sarcomas, 31 patients were identified with a primary leiomyosarcoma of bone. There were 12 males and 19 females with a mean age of 46 and tumour size of 8 cm. The most common site was the distal femur followed by the proximal tibia. Treatment was with chemotherapy and surgical resection. Seven of the patients had metastases at diagnosis. Surgery was carried out in 28 patients, 8 having amputation and 20 limb salvage. Three patients developed local recurrence, but half developed metastases. All patient disease-specific survival was 57% at five years and 44% at 10 yrs but for those without metastases was 82% and 60%, respectively. The only prognostic factors were metastases at diagnosis. *Conclusion*. Leiomyosarcoma of bone is a very rare primary malignant bone tumour affecting a predominantly older population. Despite the high incidence of metastases, survival is better than for other bone sarcomas for those without metastases at diagnosis.

## 1. Introduction

Primary malignant bone tumours are rare with an incidence of approximately 9 million population per year. The most common ones are osteosarcoma, Ewing's sarcoma, and chondrosarcoma, but a variety of other smaller groups exist, some with specific features (e.g., chordoma) but others have a variety of different histotypes. Over the years these have been categorised variously as fibrosarcoma, MFH, and spindle cell sarcoma, but recent immunohistochemical methods have sometimes allowed more specific characterisation.

Leiomyosarcomas (LMSs) are usually malignant soft tissue tumours with smooth muscle differentiation apparent with immunohistochemical or electron microscope studies (WHO). They can also occur as primary in bone, or secondary metastasising from a distant source (e.g., a primary leiomyosarcoma either of soft tissue or sometimes the uterus). They were first described in 1965 by Evans and Sanerkin [1] in the proximal tibia of a 73-year-old man and have since been slowly accepted in published data as a unique classification described in the literature by series of small case studies. To our knowledge, around 90 cases have been described with the largest by Antonescu et al. in 1997 [2].

We have reviewed our experience of treating patients with primary leiomyosarcoma of bone between 1989 and 2011, trying to identify prognostic indicators and outcomes.

## 2. Patients and Methods

All patients diagnosed with a primary leiomyosarcoma of bone, treated at the Royal Orthopaedic Hospital, Birmingham were included in the study from 1970 to 2011. 35 patients were identified from the database of 3364 with a primary bone sarcoma (<1%). Four patients were excluded as they were only referred for advice or had no treatment at our centre. All patients underwent complete staging with bone scans and CT chest with MRI of the affected bone after 1992. In female patients, the possibility of this being a metastasis from a uterine leiomyosarcoma was excluded by history and appropriate scans. Throughout this time period, patients were treated in the same manner as patients with osteosarcoma, receiving chemotherapy if they had a high grade tumour and were under the age of about 60. Patients with low-grade tumours or much over the age of 60 had no chemotherapy but had surgical resection aiming to achieve clear margins. Throughout this time period, the most common chemotherapy drugs used were cisplatin and adriamycin. 

Data was collected from the prospectively compiled database, and further information was obtained from the medical records. Patient, tumour, treatment, and outcome data was recorded. This included year of diagnosis, grade, Enneking stage, site, biopsy type, presence of fracture at presentation, size, surgical procedure, surgical and pathological margins, chemotherapy, presence of metastasis at diagnosis and during follow up, and date/cause of death if applicable. Treatment details included the initial treatment (surgery versus chemo), the type of surgical intervention, the surgical and pathological margins, and the timing/regime of chemotherapy. Follow-up data included the time to local recurrence, time to metastasis, time to last followup if alive, or time to death.

Statistical analysis was carried out using Statview. Kaplan-Meier survival curves were used to show survival, and univariate analysis with Cox regression was used to try and identify potential prognostic factors. Significance was taken with a *P* value < 0.05.

## 3. Diagnostic Features

**Figure 1:**
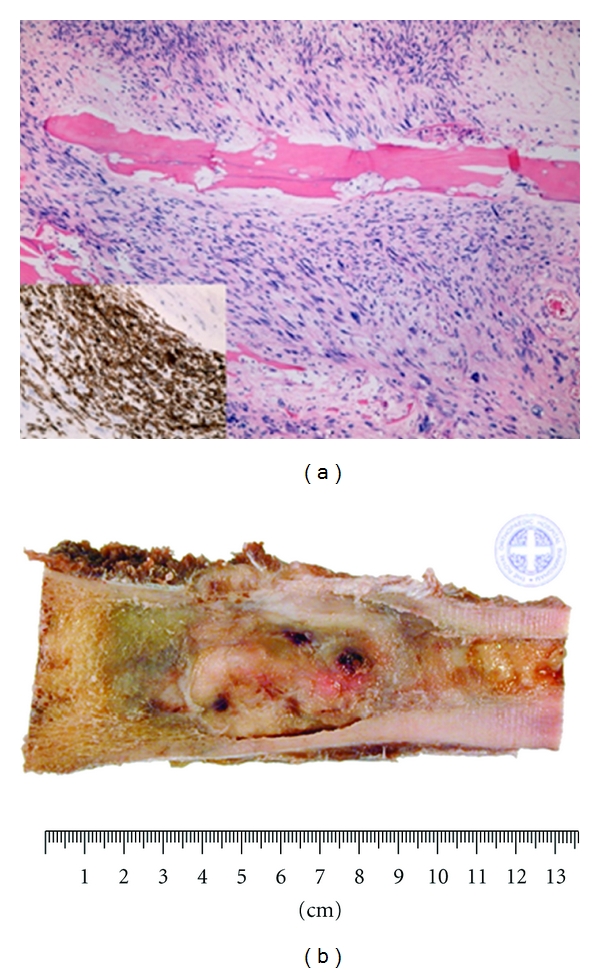
(a) Microscopic appearance of leiomyosarcoma-pleomorphic spindle cells with atypical cells, (b) typical macroscopic fleshy white appearance with 3 foci of necrosis of a leiomyosarcoma.

Primary leiomyosarcoma of bone shows smooth muscle differentiation with immunohistochemical studies. Macroscopically, these lesions appear as fleshy, greyish white tumours with areas of necrosis (Figure 1). Histologically, the features are similar to leiomyosarcoma of any other site and are characterised by fascicles of plump pleomorphic spindle cells which show marked cytological atypia (Figure 1). The tumour lacks osteoid. Immunohistochemically, the cells show positive staining with smooth muscle actin, desmin, and caldesmon. The diagnosis of leiomyosarcoma of bone was made in all cases on the basis of the above criteria applied retrospectively for cases diagnosed earlier in the series when desmin was not available. If the resection specimen contained osteoid, then the tumour was reclassified as an osteosarcoma. Cases where a soft-tissue leiomyosarcoma invaded bone secondarily were excluded.

## 4. Results

**Figure 2:**
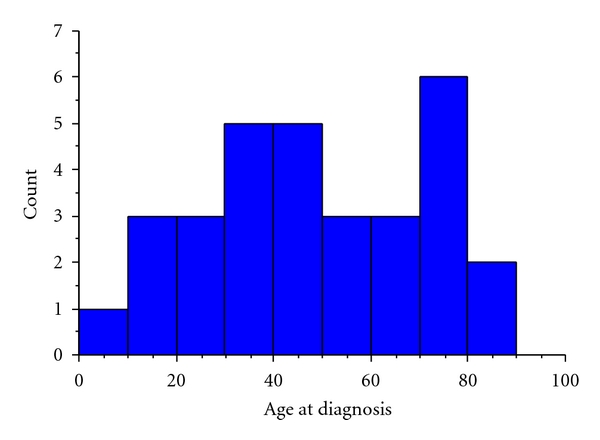
Histogram to show the distribution of age at diagnosis.

Of the 31 eligible patients identified from the oncology database, 12 were male and 19 female (39% : 61% male : female). The median age at diagnosis was 46 years old, ranging from 9 to 88 years (Figure 2).

The most common site affected was the distal femur in 14 patients (45%), followed by proximal tibia in 8 patients (26%), and proximal humerus in 2 patients (6%). Other sites affected included the mid-shaft of the clavicle, distal humerus, proximal fibula, ileum, blade of scapula, and distal tibia, each in 1 patient (3%). 13 of 31 (42%) had a pathological fracture, four of whom had it fixed before the diagnosis was made.

The median size of the tumour at diagnosis was 8 cm. Five were low-grade tumours (16%) and 26 were of high grade. Five patients were staged as Enneking stage 1b, four were stage 2a, and 15 were 2b. Six patients had metastases at the time of diagnosis (stage 3); four patients (13%) had deposits in the lungs, while one patient had metastases in the lymph nodes, and one in a distant bone.

18 of the 31 patients (58%) had chemotherapy, with 17 of those 18 having neoadjuvant chemotherapy. Only 3 of 17 patients had >90% necrosis in the resected specimen. 30 of the 31 patients (97%) had surgical resection with eight having amputations, two bone resections and 20 having endoprosthetic replacements. Surgical margins were wide in 24 (80%), marginal in five and intralesional in one.

Local recurrence occurred in three patients. This was not statistically related either to the margins of excision, or the presence of a pathological fracture, or the response to chemotherapy. Two of the three subsequently developed metastases and died.

**Figure 3:**
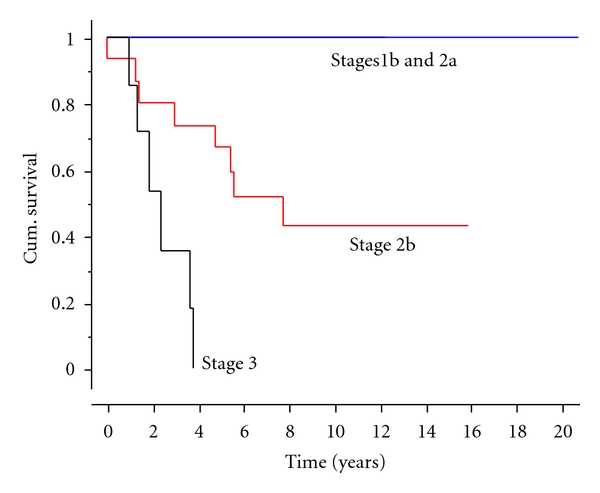
Kaplan-Meier plot indicating survival by the stage of leiomyosarcoma.

The overall survival rate was 62% at five years (Figure 3). The survival rate and prognosis were closely related to stage of diagnosis; all patients with stage 1 or 2a surviving, patients with stage 2b tumours had a 60% survival at 5 years, 43% survival at 10 and 15 years, and patients with stage 3 tumours had a median survival of 2 years, but all had died within four years.

**Figure 4:**
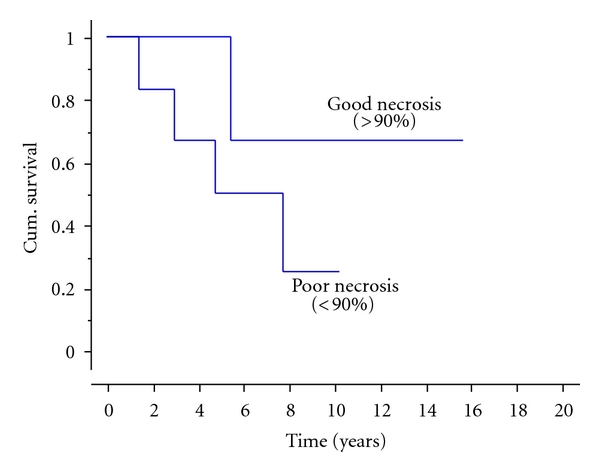
Kaplan-Meier plot indicating result of chemotherapy necrosis on survival (*P* = 0.3).

The response to chemotherapy was analysed in the 15 patients with stage 2b tumours who had chemotherapy. Although not statistically significant, those whose response to chemotherapy showed a greater than 90% necrosis appeared to have a better prognosis (Figure 4).

Statistically the only factor associated with improved survival was the absence of metastases at diagnosis (*P* = 0.0053).

## 5. Discussion

Preexisting data on primary leiomyosarcoma is limited simply due to the low incidence of this rare tumour. Historically, since the discovery and diagnosis of this type of tumour as a separate entity in 1965 [1], most cases have been presented as small case series [3–10] leading to difficulty in extrapolating results to the whole patient population.

Adelani et al., (2009) [3] found the median age affected to be 47 years, with a range from 9 to 87 years, while the median age in our series was 46, ranging from 9 to 88 years. Generally, it is felt that there is an equal distribution between the sexes for primary leiomyosarcomas [2, 7, 8]; however, our series reflects a slight male predominance, as do several other smaller studies [9, 11, 12]. In terms of tumour locality, our cohort agreed with the consensus that long bones are primarily affected, predominantly the distal femur and proximal tibia with 45% and 26% of patients, respectively [2, 5, 13, 14]. Interestingly, other studies have found the craniofacial skeleton to be the second most common area affected, while none of our patients had skull tumours, this most likely reflecting our local referral practice [15–19].

Surgical excision with wide margins remains the gold standard for curative management, amputation usually being reserved for tumours surrounding the neurovascular bundle, or with extensive soft tissue involvement. In our series, the majority of those surgically treated had limb salvage (73%), the relatively high amputation rate (8 of 30, 27%) being largely due to pathological fractures in four and extensive soft tissue involvement in the others. The role of preoperative chemotherapy remains debatable, [2, 3] reflected by our cohort; 58% received chemotherapy, and only 18% achieved >90% necrosis (this being considerably less than the 36% for patients treated for osteosarcoma over the same time period) [20]. Although not statistically significant, those patients who did respond well to chemotherapy had a trend towards better survival.

Many of the case series published have reflected a poor prognosis, although no one has successfully stratified the risk according to stage/grade. Antonescu et al. produced the largest series to our knowledge [2], presenting 33 patients with primary leiomyosarcoma between 1977 and 1996. They had an average follow-up period of 30 months and found no significant differences in disease free of overall survival rates between low- and high-grade tumours. In that series, only 21% had neoadjuvant chemotherapy, and there was no clear difference in survival between those treated with chemotherapy and those without. More recently, Rekhi et al. [10] presented a series of 8 cases, none of whom had chemotherapy. All the patients went on to develop metastases within 12 months following the original diagnosis leading the authors to conclude that it was a dismal condition to have.

This study has shown that prognosis is based on the stage of diagnosis; with Enneking stages 1b or 2a cases achieving 100% survival, stage 2b tumours having a 60% survival at 5 years and 43% survival at 10 and 15 years. These figures are certainly comparable with survival of patients treated with osteosarcoma over the same time period which averaged 56% for patients with high-grade nonmetastatic disease [21].

In conclusion, primary leiomyosarcomas of bone are aggressive tumours which should be treated just like osteosarcoma but which, in our experience, have a slightly better prognosis than patients with osteosarcoma. The optimum chemotherapy regime, like for osteosarcoma, remains to be established, but surgically they should be excised with clear margins, like other bone sarcomas.
